# Rethinking diaphragmatic ultrasound for AECOPD weaning prediction: considerations on parameter selection, thresholds, and outcome definitions

**DOI:** 10.1186/s13054-026-06398-8

**Published:** 2026-10-15

**Authors:** Qianqian Xie, Meiqin Yuan, Yan Sun

**Affiliations:** Yantai Yuhuangding Hospital Affiliated to Qingdao University, No. 20 Yuhuangding East Road, Yantai, Shandong 264000 China

Dear Editor,

We read with interest the systematic review and meta-analysis by Zhou and colleagues on the predictive value of diaphragmatic ultrasound for weaning outcomes in mechanically ventilated patients with acute exacerbation of chronic obstructive pulmonary disease (AECOPD) [1]. By integrating four diaphragmatic parameters within a disease-specific population, this work provides clinically relevant evidence.

We write to raise a point regarding the consistency of the reference standard used to define “weaning failure.” As the authors acknowledge, the 23 included studies operationalized weaning failure inconsistently: some used failure of the spontaneous breathing trial (SBT) as the endpoint, others used reintubation within 48–72 h after extubation, and still others counted initiation of non-invasive ventilation, high-flow nasal oxygen, or death within varying follow-up windows as failure [1]. These events are not biologically equivalent: SBT failure reflects intolerance of spontaneous ventilation, whereas post-extubation reintubation/NIV reflects failure of airway protection and secretion clearance [2]. Pooled under the single label of “weaning failure,” they may systematically dilute or distort the association between each ultrasound parameter and the endpoint.

We note that the authors included “definition of weaning failure” as a study-level covariate in their univariate meta-regression and reported no significant association with the diagnostic performance of DE, DTF, or D-RSBI. We would like to raise three further points.

First, we appreciate that the authors have already discussed this heterogeneity in the published article [1], which is consistent with prior calls for standardized weaning outcome classification such as the WIND classification [3]. We would further emphasize that, because the eligibility criteria required a binary weaning outcome, the pooled estimates cannot distinguish between easy weaning, difficult weaning, and prolonged weaning. Although a WIND-stratified analysis is not feasible within the present dataset, we suggest explicitly stating this interpretive limitation in the discussion: the pooled sensitivity and specificity reflect a broad outcome that mixes several weaning trajectories rather than a specific weaning phenotype.

Second, DCV was evaluated in only four cohorts and was not entered into meta-regression; D-RSBI, with eight cohorts, likewise offers limited statistical power. With such small samples, a negative meta-regression result for the reference-standard covariate should be interpreted as an absence of evidence rather than evidence of absence.

Third, the authors did not perform a stratified subgroup or sensitivity analysis by reference-standard type (SBT failure versus post-extubation reintubation/NIV). We understand that “weaning-failure definition” was entered as a covariate in meta-regression. However, meta-regression and stratified subgroup analysis are not equivalent: the former tests a linear association between a study-level covariate and the pooled effect, whereas the latter directly compares pooled estimates across reference-standard strata. Where the reference standard may itself alter the diagnostic target, stratified analysis is more informative than meta-regression for establishing robustness.

We therefore ask whether the authors could add a stratified subgroup or sensitivity analysis by reference-standard definition, to test whether the pooled effects of DE, DTF, and D-RSBI remain consistent across definitions of weaning failure. If a given parameter performs consistently only under the stricter endpoint of post-extubation reintubation, its role in weaning decisions may need to be reconsidered. 

## Data Availability

No datasets were generated or analysed during the current study.
